# Comparative analysis of cerebrospinal fluid from the meningo-encephalitic stage of *T. b. gambiense* and *rhodesiense* sleeping sickness patients using TMT quantitative proteomics

**DOI:** 10.1016/j.dib.2015.06.011

**Published:** 2015-07-02

**Authors:** Natalia Tiberti, Jean-Charles Sanchez

**Affiliations:** Translational Biomarker Group, Department of Human Protein Sciences, University of Geneva, Geneva, Switzerland

**Keywords:** Quantitative mass spectrometry, Tandem Mass Tag – TMT, Sleeping sickness, Cerebrospinal fluid

## Abstract

The quantitative proteomics data here reported are part of a research article entitled “Increased acute immune response during the meningo-encephalitic stage of *Trypanosoma brucei rhodesiense* sleeping sickness compared to *Trypanosoma brucei gambiense”*, published by Tiberti et al., 2015. *Transl. Proteomics* 6, 1–9.

Sleeping sickness (human African trypanosomiasis – HAT) is a deadly neglected tropical disease affecting mainly rural communities in sub-Saharan Africa. This parasitic disease is caused by the *Trypanosoma brucei* (*T. b.*) parasite, which is transmitted to the human host through the bite of the tse-tse fly. Two parasite sub-species, *T. b. rhodesiense* and *T. b. gambiense*, are responsible for two clinically different and geographically separated forms of sleeping sickness.

The objective of the present study was to characterise and compare the cerebrospinal fluid (CSF) proteome of stage 2 (meningo-encephalitic stage) HAT patients suffering from *T. b. gambiense* or *T. b. rhodesiense* disease using high-throughput quantitative proteomics and the Tandem Mass Tag (TMT^®^) isobaric labelling. In order to evaluate the CSF proteome in the context of HAT pathophysiology, the protein dataset was then submitted to gene ontology and pathway analysis. Two significantly differentially expressed proteins (C-reactive protein and orosomucoid 1) were further verified on a larger population of patients (*n*=185) by ELISA, confirming the mass spectrometry results. By showing a predominant involvement of the acute immune response in *rhodesiense* HAT, the proteomics results obtained in this work will contribute to further understand the mechanisms of pathology occurring in HAT and to propose new biomarkers of potential clinical utility. The mass spectrometry raw data are available in the Pride Archive via ProteomeXchange through the identifier PXD001082.

**Table:** 

**Specifications table**	
Subject area	*Biomedicine*
More specific subject area	*Quantitative proteomics, sleeping sickness, clinical biomarkers*
Type of data	*Table of proteins significantly differentially expressed* (*obtained with Isobar*); *list of all protein identifications and quantifications* (*Excel file, Supplementary MS data*)
How data was acquired	*NSI-LTQ Orbitrap Velos* (*Thermo Electron*)
Data format	*MS/MS data analysed for identification and quantification*
Experimental factors	*CSF samples were submitted to the modified single centrifugation sample preparation for sleeping sickness diagnostic purposes, as described by Miezan et al.*[1]. *For each patients, 60 µL of CSF was used for the proteomics sample preparation.*
Experimental features	*CSF samples were analysed by quantitative proteomics. Proteins were digested with trypsin and labelled with the TMT 6-plex reagents. The pooled labelled sample was subjected to off-gel electrophoresis (OGE) fractionation* (*12 fractions*) *prior to LC–MS/MS analysis of each individual fraction.*
Data source location	S*amples were collected in the Democratic Republic of the Congo and in Uganda. All the analyses* (*quantitative proteomics, data analysis*) *have been performed in Geneva* (*Switzerland*).
Data accessibility	*Data are publicly available via ProteomeXchange and can be accessed through the identifier PXD001082* (http://www.ebi.ac.uk/pride/archive/projects/PXD001082)

## 1 Value of the data

• The TMT quantitative approach is a powerful tool to characterise and compare CSF proteome.
• The combination of proteomic and bioinformatics is useful to understand HAT pathophysiology.
• Quantitative proteomic showed that *T. b. rhodesiense* strongly evokes innate immunity activation.

## 2 Experimental design, materials and methods

### 2.1 Experimental design

**Fig. 1:**
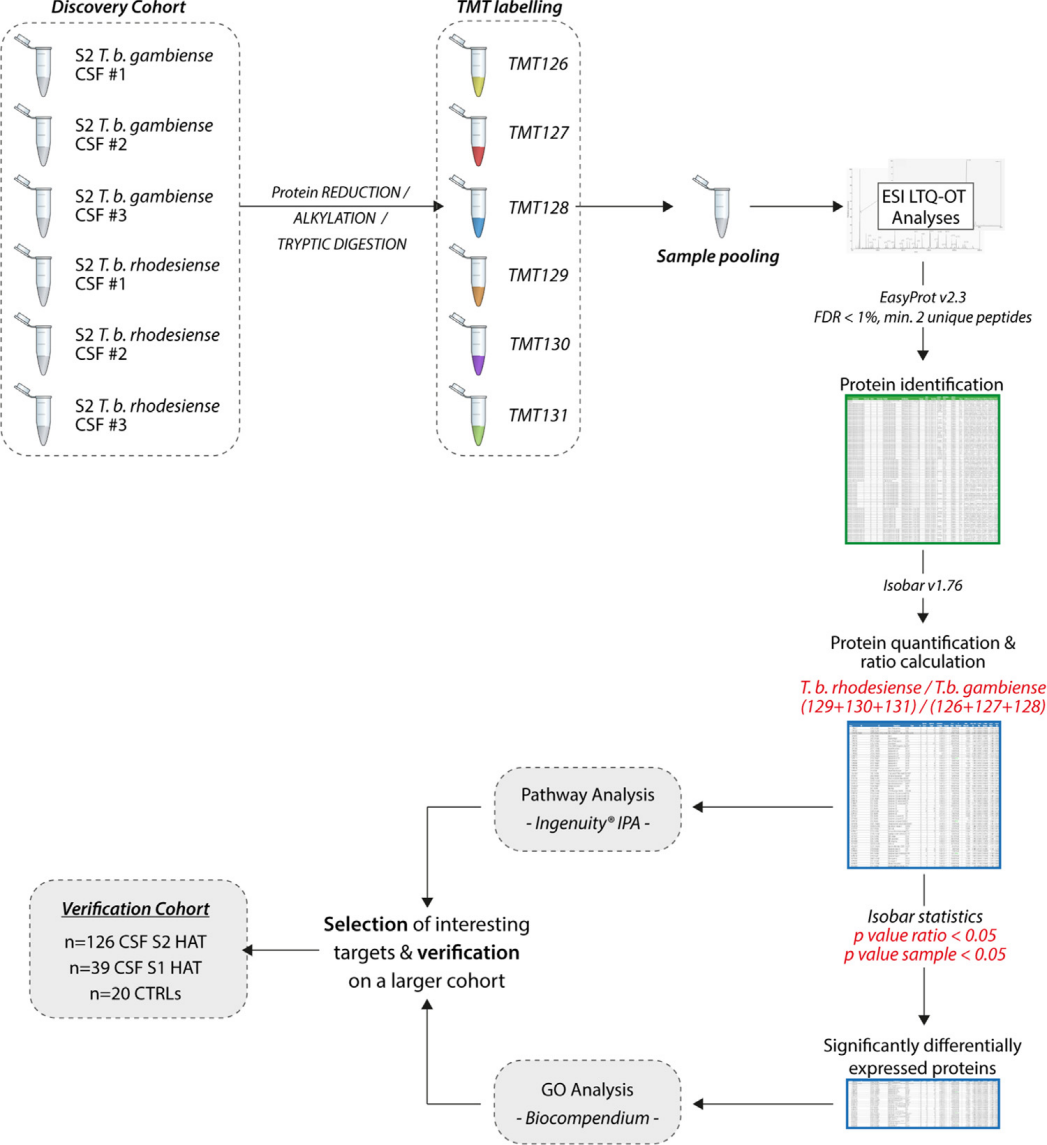
Graphical representation of the complete experimental design of the study involving the TMT 6-plex experiment and dataset described.

In the present work we investigated the cerebrospinal fluid (CSF) from *T. b. gambiense* (*n*=3) and *T. b. rhodesiense* (*n*=3) HAT patients using TMT quantitative proteomics. The quantitative analyses, performed on the identified proteins, highlighted proteins differentially expressed between the two forms of HAT. The proteomics data here presented, are part of a larger investigation on the biological mechanisms and pathways specifically associated to the *rhodesiense* form of HAT when compared to the *gambiense* one, published in 2015 [2]. The complete experimental design involving the proteomics investigations here reported is represented in Fig. 1.

### 2.2 Patients and ethical statement

HAT patients investigated by quantitative proteomics were enrolled in the Democratic Republic of the Congo (D.R.C.) and in Uganda, as part of prospective studies already published elsewhere [3,4]. The relevant Institutional and National Ethics Committees of the D.R.C., Uganda and Belgium approved the respective studies. All participants signed a written informed consent, accepted to be enrolled in the studies and had the possibility to withdraw at any moment.

Patients were diagnosed and staged for sleeping sickness according to the guidelines of the national sleeping sickness control programs of the country of sample collection. For the present study, patients were classified as stage 2 (i.e., presence of parasites in CSF and/or CSF WBC count >5 cells/µl) following WHO guidelines [5].

CSF was collected to determine HAT stage and was subjected to the modified single centrifugation for parasite detection [1]. Our proteomics analyses were performed on the supernatant of this centrifugation. All CSF samples here investigated were collected before treatment administration.

### 2.3 Proteomics

#### 2.3.1 Samples

CSF samples (*n*=6) obtained from 6 HAT patients were investigated. Among them, 3 patients suffered from S2 *T. b. gambiense* HAT and originated from the D.R.C. [3] and 3 patients suffered from S2 *T. b. rhodesiense* and originated from endemic regions in Uganda – Serere district (FINDTRYP study) [4]. The demographic description of the patients is reported in Table 1.

#### 2.3.2 Sample preparation and peptide labelling

For each sample, 60 µL of CSF was used. An internal control was spiked in each sample (0.5 µg of bovine beta-lactoglobulin, SigmaAldrich). Proteins were then reduced with 50 mM *tris*(2-carboxyethyl)phosphine (TCEP) and alkylated with 400 mM iodoacetamide, prior to digestion into peptides with trypsin 0.2 µg/µL (Promega).

Digested samples were labelled with the TMT^®^ 6-plex tagging reagents (Thermo Fisher Scientific) following manufacturer׳s instructions. *T. b. gambiense* samples were labelled with the tags TMT-126, TMT-127 and TMT-128, while *T. b. rhodesiense* samples were labelled with the tags TMT-129, TMT-130 and TMT-131 (Table 1). After tagging, the 6 samples were pooled, dried under vacuum and desalted with C18 Macro Spin Columns (Harvard Apparatus).

The pooled sample was then fractionated by off-gel electrophoresis (OGE – Agilent) into 12 fractions using a 13 cm, pH 3–10 linear IPG strip (GE Healthcare). Each OGE fraction was desalted with C18 Micro Spin Columns (Harvard Apparatus), dried under vacuum and analysed by tandem mass spectrometry [6,7].

#### 2.3.3 Mass spectrometry analyses

MS analyses were performed on a nanoelectrospray ionisation (NSI) LTQ Orbitrap (OT) velos from Thermo Electron equipped with a NanoAcquity system from Waters. Peptides were trapped on a home-made 5 µm 200 Å Magic C18 AQ (Michrom) 0.1×20 mm^2^ pre-column and separated on a home-made 5 µm 100 Å Magic C18 AQ (Michrom) 0.75×150 mm^2^ column with a gravity-pulled emitter. A gradient of 65 min was applied for the analytical separation using H_2_O/formic acid (FA) 99.9%/0.1% as solvent A and CH_3_CN/FA 99.9%/0.1% as solvent B. The gradient was run at a flow rate of 220 nL/min as follows: 0–1 min 5% B, to 35% B at 55 min and then to 80% B at 65 min.

For MS survey scans, the OT resolution was set to 60,000 and the ion population was set to 5×10^5^ with an *m/z* window from 400 to 2000. A maximum of 3 precursors were selected for both collision-induced dissociation (CID) in the LTQ and higher energy collision dissociation (HCD) with analysis in the OT. For MS/MS in the LTQ, the ion population was set to 7000 (isolation width of 2 *m/z*) while for MS/MS detection in the OT, it was set to 2×10^5^ (isolation width of 2.5 *m/z*), with resolution of 7500, first mass at *m/z*=100 and maximum injection time of 750 ms. The normalised collision energies were set to 35% for CID and 60% for HCD [7].

#### 2.3.4 Protein identification and quantification

Protein identification was obtained with the EasyProt platform v2.3 [8]. After peak list generation using ReadW software, CID and HCD spectra were merged to obtain the simultaneous identification and quantification [7].

Proteins were identified by searching peptide spectral matches against the Swiss-Prot/UniProt database (Version 13-June-2012, 536′489 entries) choosing the *Homo sapiens* taxonomy. For EasyProt protein search, carbamidomethylation of cysteines, TMT six-plex amino-termini and TMT six-plex lysines were set as fixed modifications, while oxidised methionines as variable. Trypsin was selected as the digestion enzyme with only 1 missed-cleavage allowed, only peptides with a minimum of 6 residues were selected for identification and the precursor ion tolerance was set to 10 ppm.

The efficient technical performance of the experiment was evaluated through the peptide labelling rate (over 93%) and the technical variability through the peptide relative intensity distribution of the bovine beta-lactoglobulin among the 6 channels (CV<17%).

Only proteins identified with at least 2 unique peptides and with an FDR≤1% (computed at the PSM level) were considered for further quantitative analyses using Isobar quantification tool (version 1.76) [9], embedded in EasyProt. Only peptides specific for a unique entry in the job were taken into account for protein quantification. The isotopic purity correction (according to the algorithm given by the manufacturer) of each channel and the Isobar default normalisation [9] were applied. Finally, the protein ratio *T. b. rhodesiense*/*T. b. gambiense* was computed according to the tagging design, i.e. TMT129+130+131/TMT126+127+128 and the ratio and sample *p*-Values were calculated by the software [9]. Proteins having both ratio *p*-Value (estimator of ratio accuracy relative to the quality of the spectra) and sample *p*-Value (estimator of the biological variability) significant were considered as differentially expressed. The list of proteins significantly differentially expressed is reported in Table 2.

The mass spectrometry raw data were deposited into the ProteomeXchange Consortium (http://proteomecentral.proteomexchange.org) [10] via the PRIDE partner repository [11] with the dataset identifier PXD001082.

The list of identified (*n*=239) and quantified (*n*=222) proteins, with the respective protein ratio and statistics is reported in Supplementary MS data (Tables S1 and S2).

#### 2.3.5 Gene ontology and pathway analyses

The experimental protein dataset was then evaluated in the context of HAT pathophysiology. To point out specific mechanisms significantly associated to one of the two forms of sleeping sickness, quantified proteins and proteins significantly differentially expressed were submitted to pathway (IPA Ingenuity) and gene ontology (GO – BioCompendium) analyses, respectively.

## Conflict of interest

Conflicts of interest: none.

## Figures and Tables

**Table 1:** Demographic description of patients investigated in the TMT 6-plex experiment.

	*T. b. gambiense* - D.R.C.			*T. b. rhodesiense* - UGANDA		
	**Pt #1**	**Pt #2**	**Pt #3**	**Pt #4**	**Pt #5**	**Pt #6**

Sex	F	F	F	F	F	F
Age	21	29	51	22	30	53
T+CSF	Yes	Yes	Yes	Yes	Yes	Yes
WBC/µL	135	217	97	119	231	70
**TMT tag**	**126**	**127**	**128**	**129**	**130**	**131**

T+ CSF: presence of parasites in the CSF, WBC/µL: number of leucocytes/µL of CSF.

**Table 2:** List of proteins significantly differentially expressed between *rhodesiense* and *gambiense* S2 CSF. Only proteins identified with at least 2 unique peptides and FDR<1% were considered for quantification.

**#**	**AC**	**Protein**	**Gene**	**Peptide count**	**Spectral count**	**Ratio**⁎	*p* Value ratio	***p*****Value sample**
1	P06311	Ig kappa chain V–III region IARC/BL41	Na	2	2	0.69	0.007	0.002
2	O00584	Ribonuclease T2	RNASET2	3	3	0.69	0.006	0.019
3	Q9NPR2	Semaphorin-4B	SEMA4B	2	2	0.72	0.048	0.017
4	P04114	Apolipoprotein B-100	APOB	4	5	1.36	0.041	0.045
5	P19652	Alpha-1-acid glycoprotein 2	ORM2	5	10	1.49	0.005	0.017
6	Q03591	Complement factor H-related protein 1	CFHR1	1	1	1.52	0.023	0.036
7	P02748	Complement component C9	C9	8	10	1.78	0.006	0.003
8	Q14624	Inter-alpha-trypsin inhibitor heavy chain H4	ITIH4	10	18	1.80	0.005	0.026
9	P18428	Lipopolysaccharide-binding protein	LBP	2	2	2.31	0.046	0.000
10	P02763	Alpha-1-acid glycoprotein 1	ORM1	6	28	2.69	0.001	0.000
11	P02741	C-reactive protein	CRP	3	5	9.30	0.001	0.000

Peptide count and spectral count correspond to the number of peptides and of spectra used for the quantification, respectively.

⁎ *T. b. rhodesiense*/*T. b. gambiense* [129+130+131/126+127+128].
